# The role of direct current potentials in assessment of energy metabolism pattern in surgical patients

**DOI:** 10.1186/2197-425X-3-S1-A577

**Published:** 2015-10-01

**Authors:** I Zabolotskikh, T Musaeva, K Zybin

**Affiliations:** Kuban Medical University, Krasnodar, Russian Federation

## Introduction

Correction of metabolic disorders is an obligatory component in treatment of the majority of the pathological processes, allowing to avoid deterioration of a condition of the patient and accordingly to reduce time of stay in a hospital.

## Objectives

Develop a mode of noninvasive, informative and accessible in practical application the express-test of energy metabolism pattern

## Methods

The study was performed on 183 patients underwent abdominal surgery (duration 6-9 hours). Were assessed central hemodynamics, gas exchange, temperature and nutrition state for the nutrition support. The nutritional support has based on monitored nutritional status. Depending on energy metabolism pattern defined by biochemical tests, patients were divided on 4 groups: group 1 - enzymic energy metabolism pattern, group 2 - hypoxic energy metabolism pattern, group 3 - substrate energy metabolism pattern, group 4 included the patients with hypermetabolic energy metabolism pattern. The concurrent registration of direct-current potential (DCP) and number of superslow oscillations (SSO) was made in a forehead-palm lead.

## Results

The enzymic energy metabolism pattern correspond with zero to 3 SSO and DCP value more than -10 mV; the hypoxic energy metabolism pattern correspond with more than 10 SSO and DCP value more than -15 mV; the substrate energy metabolism pattern correspond with more than 10 SSO and “optimal” DCP value of -15 to -35 mV; the hypermetabolic energy metabolism pattern correspond with more than 18 SSO and DCP value less than -35 mV; optimal (normal) energy metabolism pattern correspond with 4 to 9 SSO and DCP value of -15 to -35 mV.

## Conclusions

The DCP monitoring allows to determine the kind of energy metabolism pattern on the basis of number of superslow oscillations of direct current potential during ten minutes and background values of direct-current potential.
